# CAR T-Cell Therapy: On the Verge of Breakthrough in Many Hematologic Malignancies

**DOI:** 10.6004/jadpro.2017.8.3.3

**Published:** 2017-04-01

**Authors:** Edward Stadtmauer, Patricia A. Mangan

**Affiliations:** University of Pennsylvania Abramson Cancer Center, Philadelphia

## Abstract

There have been promising results from clinical trials on the efficacy of engineering patients’ immune cells to treat their cancers. CAR T-cell therapy also has unique toxicities advanced practitioners should be aware of.

Chimeric antigen receptor (CAR) T-cell therapy is being studied mainly for the treatment of B-cell malignancies, but preliminary work is underway for its use in brain cancer, breast cancer, pancreatic cancer, mesothelioma, and others.

The rationale for the use of CAR T-cell therapy is that refractoriness or relapse observed with conventional chemotherapy and immunotherapy is common, limiting survival, said Edward Stadtmauer, MD, Chief, Hematologic Malignancies Section, University of Pennsylvania Abramson Cancer Center, Philadelphia.

The philosophy behind CAR T-cell therapy is to genetically modify autologous T cells with redirected specificity to tumor antigens. This process produces a graft-vs.-tumor effect without the graft-vs.-host disease that occurs with allogeneic stem cell transplant, he said at the 2016 JADPRO Live conference.

The advantages of genetically modified autologous T cells are their specificity, the amplified response they induce, and the memory activity generated "to keep eating up any potential tumor that comes back," said Dr. Stadtmauer.

The best target for T-cell therapy is one that is expressed uniquely on the tumor cells. "These cells can sometimes be so active and so aggressive that if, by chance, the heart has the same target as the tumor, these cells will not only attack the tumor but attack the heart," he said.

CD19 was chosen as the first target for several reasons. CD19 is expressed on the surface of most B-cell malignancies. The earliest B cells are CD19-positive; these are the cells that lead to acute lymphocytic leukemia (ALL) and, as they mature, lead to chronic lymphocytic leukemia (CLL) and lymphomas. Antibodies against CD19 inhibit the growth of tumor cells (see Figure 1).

**Figure 1:**
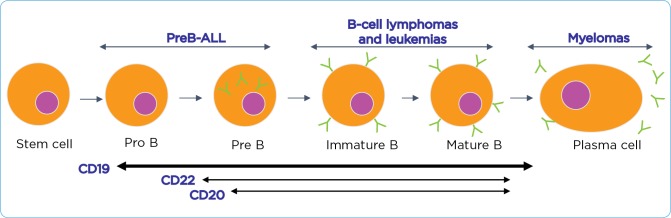
CD19 is an ideal tumor target. ALL = acute lymphoblastic leukemia. Information from Scheuermann et al. (1995), adapted from Janeway et al. (2001).

## COMPONENTS OF CARs

Adoptive T-cell therapy using CARs involves harvesting lymphocytes from the patient’s blood by apheresis. The CAR has three components: (1) an ectodomain, which is an antigen recognition composed of light chains and heavy chains directed against the target, such as CD19; (2) a transmembrane domain, traversing the lipid bilayer; and (3) an endodomain, which consists of stimulatory and costimulatory molecules, such as CD28 and 4-1BB, which send a signal to the cell to proliferate and activate once the CAR is attached.

"The first thing that had to be done was create a receptor and get the DNA [to code for the CAR]," said Dr. Stadtmauer. "The next step was to insert this DNA into one’s T cell. The vehicle that we used for this is a lentiviral vector; so in effect, we infect these cells with a virus. It does what viruses do: insert genetic material into the T cell."

**Figure 2:**
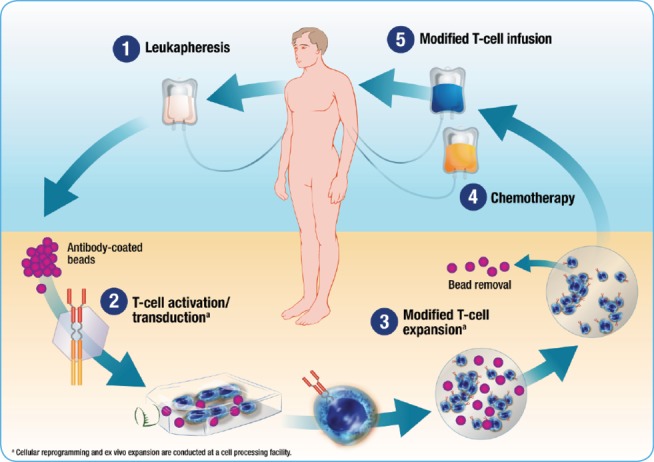
Overview of activated/engineered T-cell therapy. Image courtesy of DL Porter.

Outside the body, the T cells then are incubated with an artificial dendritic cell, which is a magnetic bead coated with anti-CD3 and anti-CD28 antibodies, which makes the T cells proliferate and become activated. Before CAR T-cell infusion, patients undergo lymphodepletion therapy to allow the T cells to engraft (see Figure 2).

## CLINICAL TRIAL RESULTS: ANTI-CD19 CAR T-CELL THERAPY

Since 2011, results have been published from 8 clinical trials of CD19-targeted CAR T cells for the treatment of B-cell malignancies, and there are at least 27 ongoing/planned trials at 10 centers (Davila et al., 2014; Porter et al., 2013; Maus, Grupp, Porter, & June, 2014).

Responses have been observed in heavily pretreated patients with CLL, ALL, and B-cell non-Hodgkin lymphoma (NHL). In CLL, the overall response rate (ORR) has been 40% to 50%, with ORRs of 80% to 90% in the treatment of relapsed/refractory ALL (Maude et al., 2014). Some patients have had a complete response (CR) extending beyond 3 years. Anti-CD19 CAR T-cell therapy (CTL019) has been granted Breakthrough Designation for ALL by the US Food and Drug Administration (FDA).

In a clinical trial (started in 2014) of patients with relapsed/refractory CD19-positive NHL who received a median of three prior lines of therapy, lymphodepletion was followed by CTL019 infusion in 43 patients (Schuster et al., 2015). In the 15 evaluable patients with diffuse large B-cell lymphoma (DLBCL), the ORR at 3 months was 47% (3 patients with CR and 4 with partial response [PR]). Three of the patients with PRs at 3 months converted to CRs by 6 months; one patient with a PR at 3 months had progressive disease at 6 months.

With a median follow-up of 17.3 months, no responder has had a relapse, reported Dr. Stadtmauer. "These are durable responses; that’s the key," he said.

Fourteen patients enrolled had follicular lymphoma, with a median of five prior lines of therapy. Among 13 evaluable patients, the ORR at 3 months was 77% (six with CR and four with PR). Three patients with PRs by imaging criteria at 3 months converted to CRs by 6 months, and 1 patient with a PR at 3 months, who remained in PR at 6 months and 9 months, had progressive disease at approximately 12 months. Progression-free survival was 64.1% at a median follow-up of 14.3 months.

CD19 has not been considered the most logical target for multiple myeloma because the dominant population of myeloma plasma cells is CD19-negative. However, minor CD19-positive components of the myeloma clone can be identified in patients. Myeloma stem cells can be CD19-positive, explained Dr. Stadtmauer, and evidence exists that some plasma cells can "de-evolve" into CD19-positive or chemoresistant clones, in what is called phenotypic transition states. Some studies have suggested that the less mature components of the myeloma clone may add to the clonogenic and disease-propagating potential.

"Maybe focusing on CD19 won’t kill every myeloma cell, but it might kill the mothers, and it might kill the most resistant cells," he suggested. A study was designed to confirm this hypothesis in which 10 patients with multiple myeloma and prior stem cell transplant with a duration of response < 1 year who required a salvage stem cell transplant were also infused with anti-CD19 CAR T-cell therapy using CTL019 (Garfall et al., 2015). There was no significant toxicity attributable to CTL019. One episode of grade 1 cytokine-release syndrome (CRS) was observed. The study demonstrated the feasibility of obtaining healthy T cells from heavily pretreated patients with myeloma. Three patients had remission inversions, and durable responses for up to 15 months were observed.

In addition, CAR T-cells directed against B-cell maturation antigen (*BCMA*) have been studied in multiple myeloma, because *BCMA* expression is upregulated during normal B-cell differentiation into plasma cells. So far, 12 patients with relapsed/refractory multiple myeloma have been treated at the National Institutes of Health (Ali et al., 2015). The first patient enrolled in a similar study at the University of Pennsylvania, who had 11 prior lines of therapy and whose myeloma cells expressed *BCMA*, had a CR within 4 weeks of treatment, with a negative bone marrow biopsy, and response is ongoing at 15-plus months. The patient suffered grade 3 CRS, which responded to treatment with tocilizumab (Actemra).

## MANAGING CAR T-CELL THERAPY TOXICITIES

Therapy with CAR T cells can be given over a single infusion or multiple infusions over 3 days, depending on the protocol. Fever and chills are sometimes associated with the infusions, said Patricia A. Mangan, RN, MSN, APRN-BC, nurse leader of Hematologic Malignancies, University of Pennsylvania Abramson Cancer Center, Philadelphia. Tumor lysis syndrome is a potential risk when patients with a high tumor burden are treated, but it’s not a prominent feature, she added.

Additional toxicities are those related to lymphodepleting chemotherapy and high-dose stem cell transplant (i.e., cytopenias). Profound B-cell aplasia is another risk; therefore, intravenous immunoglobulin (IVIG) levels should be monitored and support given, as necessary, to prevent recurrent infection.

The most serious risk specific to CAR T-cell infusion is CRS, she said, as dramatic elevations in cytokines including interleukin-6 (IL-6) have been shown following infusion. Almost all responding patients develop CRS. "It mimics a septic event, where we see some very high fever, hypotension, and some hypoxia," said Ms. Mangan. If therapy is administered on an outpatient basis, patients are asked to monitor their body temperature closely.

The peak activity for cytokine release is usually around day 10 after infusion, although release can occur sooner or later than this. In addition, CRS can be associated with hemophagocytic lymphohistiocytosis/macrophage activation syndrome, in which ferritin levels can exceed 50,000 ng/mL. C-reactive protein (CRP) level is a surrogate marker for IL-6 levels and can be elevated as well. Mental status changes can also occur but generally fully resolve without specific treatment.

The process can be "turned off" by steroids but with an undesirable therapeutic effect, as CAR T cells are lost. "Miraculously, we have found the antidote, and it’s the anti–IL-6 receptor monoclonal antibody tocilizumb," she said. "We have pulled people out of very serious illnesses with the use of tocilizumab."

Cytokine-release syndrome is rapidly reversed with tocilizumab, but the optimal timing of administration to abrogate CRS is not fully known. "We’ve given it as soon as day 2 and as late as day 11, or even a little later," revealed Ms. Mangan.

## POTENTIATING T-CELL THERAPY

Bispecific T-cell engagers (BiTES) are "off-the-shelf" agents that provide T cell activation without requiring collection of the patient’s own T cells. A piece of the BiTE attaches to CD3 on the surface of the T cell, where it activates the T cell. The other component of the BiTE attaches to a tumor cell protein, latches onto the target, and directs the T cell to act against tumor cells.

In the case of blinatumomab (Blincyto), the target is CD19. Blinatumomab was approved by the FDA in 2014 for the treatment of relapsed/refractory Philadelphia chromosome–negative relapsed/refractory B-cell ALL. Potential side effects of blinatumomab are flu-like symptoms and central nervous system side effects (i.e., encephalopathy, tremor, apraxia).

Moreover, CAR-T cell therapy can be potentiated with agents such as programmed cell death protein 1 (PD-1) inhibitors. This protein is an immune checkpoint regulating the immune system by preventing the activation of T cells, promoting self-tolerance. Programmed cell death protein 1 and its ligand (PD-L1) negatively regulate immune responses. Many cancer cells make PD-1, which inhibits T cells from attacking the tumor. When PD-L1 is blocked from binding with PD-1, the brake is "taken off" the T cell.

Nivolumab (Opdivo) is an anti–PD-1 monoclonal antibody approved for the treatment of melanoma, non–small cell lung cancer, and kidney cancer. Alone, it produced no objective responses in the treatment of relapsed/refractory multiple myeloma in a phase I study; however, durable stable disease was achieved in 67% of the 27 patients with relapsed/refractory disease treated with nivolumab, providing some promise that adding immunomodulatory therapy to CAR T-cell therapy may improve response (Lesokhin et al., 2014). 
